# The multimodal management of GH-secreting pituitary adenomas: predictive factors, strategies and outcomes

**Published:** 2016

**Authors:** A Buliman, LG Tataranu, V Ciubotaru, TL Cazac, C Dumitrache

**Affiliations:** * Titu Maiorescu University, Faculty of Medicine, Bucharest, Romania; ** “Carol Davila” University of Medicine and Pharmacy, Bucharest, Romania; *** “Bagdasar-Arseni” Clinical Emergency Hospital, Bucharest, Romania; **** “C.I. Parhon” National Institute of Endocrinology, Bucharest, Romania

**Keywords:** growth hormone, acromegaly, pituitary adenoma, transsphenoidal surgery, predictive factors

## Abstract

**Object.** The aim of this study was to analyze a series of 28 patients with acromegaly who underwent a multimodal surgical, medical and radiosurgical therapy, with a special attention to the advantages, complications, and predictive factors of a successful outcome.

**Methods.** 28 consecutive cases of GH-secreting pituitary adenomas, who underwent transsphenoidal endoscopic or microscopic surgery, between 1 January 2014 and 31 December 2014 were retrospectively reviewed. Tumors were classified according to the diameter, measured on MRI, as micro- or macroadenomas, and parasellar (cavernous sinus) tumor extension was analyzed based on the Knosp grading score. The mean follow-up period was of 18.4 months. Criteria justifying the complete hormonal remission were preoperative basal serum GH < 2.5 μg/ L, preoperative nadirGH < 1 ng/ L after OGTT and normal preoperative IGF–I levels age and sex-matched.

**Results.** An overall complete hormonal remission rate was achieved in 64.3% of the patients. The remission rate was higher in patients with microadenomas (77.8%) than in those with macroadenomas (57.9%). A number of predictive factors, which might have interfered with the hormonal remission rate from a statistical, clinical and paraclinical point of view, were identified: tumor size (r = 0.625), preoperative GH serum levels (r = -0.517), cavernous sinus extension was quantified according to Knosp grading score (r = 0.469) and the degree of tumor subtotal resection (r = 0.598).

**Conclusions.** Favorable hormonal and visual remission rates can be achieved after transsphenoidal resection of GH-secreting pituitary adenomas; however, the management remains challenging, the increased surgical experience being important for higher cure rates. If a biochemical hormonal cure is not achieved postoperatively, adjuvant medical or radio surgical therapy can be recommended.

## Introduction

Acromegaly is a chronic disease due to high serum levels of the growth hormone and the insulin-like growth factor-I, caused by a GH secreting pituitary adenoma. Elevated GH and IGF-I levels are considered to increase the cardiovascular, respiratory, endocrine, and metabolic morbidities [**1**-**3**].

The main goal of the surgical approach, in GH-secreting pituitary adenoma, is represented by total tumor resection, within safe conditions, avoiding major postoperative complications and preventing any recurrence [**1**-**3**]. In the multimodal management of acromegaly, first-line therapy is represented by the surgical technique, through microscopic or newly developed endoscopic approach. Gamma Knife radiosurgery and medical therapy are instituted when postoperative hormonal remission is not achieved or when serum hormone levels continue to be elevated due to a tumor residue.

Several factors have been established in literature as important predictors of surgical outcome, the early diagnose and treatment leading to high rates of remission and avoiding long-term comorbidities [**48**,**49**].

The aim of this study was to analyze a series of 28 patients with acromegaly who underwent a multimodal surgical, medical and radiosurgical therapy, with a special attention to the advantages, complications, and predictive factors of a successful outcome.

## Methods

**Patient Population**

A retrospective, observational and descriptive study was conducted on 28 patients diagnosed with growth hormone-secreting pituitary adenomas (GH), from 1 January 2014 to 31 December 2014.

The study was performed under the authorization of the ethics committee of “Bagdasar Arseni” Emergency Hospital, in Bucharest, and all the patients consented with the proposed treatment after a careful explanation of all the therapeutic options.

During our research, medical records were obtained and reviewed after a careful analysis of every patient’s observation charts, which contained medical history, clinical, biological, imaging, and therapeutic aspects.

If decreased visual acuity or fields defects were observed on the initial clinical evaluation, patients were referred for standard ophthalmological evaluation before and after surgery.

The mean postoperative follow up of patients was of 18.4 months, with a range between 12 and 24 months. 

**Endocrine Evaluation**

The biochemical hormonal investigations were conducted in laboratories associated to specialized endocrinology clinics. All patients underwent a preoperative hormonal assessment for their fasting GH and IGF- I levels and anterior pituitary function. After a basal GH and IGF-I level measurement, all the patients had an OGTT. The endocrine evaluation was repeated at 3, 6, and 12 months postoperatively and annually afterwards.

Criteria justifying a complete hormonal remission, also reported in literature, according to the latest guidelines, are represented by: a normal concentration of IGF-1 age and sex-matched, a basal serum GH less than 2.5 μg/ L, and a nadir GH level after oral glucose tolerance test of less than 1 μg/ L. A complete and early remission rate was considered if all these conditions were fulfilled.

These criteria were followed throughout the entire period of observation in order to detect the postoperative recurrences and efficiently monitor the adequate response of the chosen therapeutic method. 

**Imaging Evaluation**

Imaging investigations, reported in every patient’s observation charts were performed within the neurosurgical department of “Bagdasar-Arseni” Emergency Hospital. All the patients were evaluated both through computer tomography and magnetic resonance imaging preoperatively and repeated at 3, 6, 12 months postoperatively and annually afterwards. The MRI investigation (1,5 T, 2,5 mm slice thickness) was performed before and after Gadolinium 5mg administration, in all 3 section views: sagittal, coronal and axial. All pituitary adenomas were classified according to size and degree of extension by using the MRI contrast imaging. Tumors of less than 1 cm were classified as microadenomas, and those greater than or equal to 1 cm were catalogued as maroadenomas. Parasellar extensions were classified by using the Knosp grading score.

Angio-CT or Angio-RM were performed in patients whom were suspected of a coexisting aneurysm with sellar localization, as well as in patients with a median disposal of the intracavernos carotid arteries on the MRI. In our study, pacients previously operated through a microscopic or endoscopic transsfenoidal approach were not included.

**Treatment**

All the patients were treated exclusively through the same transnasal transsphenoidal microsurgical approach by the senior neurosurgeons (C.V. and T.L). 

The period of hosital stay ranged between 2 and 7 days, with an average of 4 days. Patients in whom a biochemical hormonal cure was not achieved postoperatively and contrast MRI showed a residual tumor after surgery have received adjuvant medical or radiosurgical therapy, taking into account their preferences. 

Gamma Knife radiosurgery was established 3 months postoperatively, when a tumor residue was visualized on the contrast MRI, responsable of high serum leves of IGF-1 and GH.

**Statistical Analysis**

Descriptive Statistics, consisting of the calculation of the average, median, standard deviation of different parameters, was realized by using Microsoft Excel Program. The statistical analysis, consisting of the Pearson linear correlations between different predictive parameters was performed by using the MedCalc Application.

## Results

**Patients**

Of all 28 patients, aged between 22 and 76 years (mean 50.7), (**Table 1**), 16 women and 12 men (Male/ Female ratio was 3:1) showed clinical and paraclinical manifestations suggestive of acromegaly.

**Table 1:** Statistical data concerning the patient’s age

	*Min*	*Max*	*Mean*	*Median*	*Stddev*
*Age (years)*	22	76	50,71429	52	14,10891

**Fig. 1:**
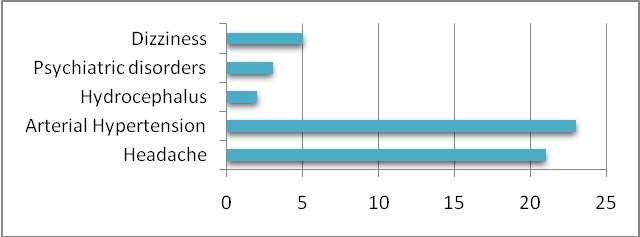
Statistical data concerning the patient’s signs and symptoms on admission

Hypertension was the main sign among the patients in our study (23 patients, 82.1%) along with headache (21 patients, 75%), sleep apnea, arthralgia (13 patients, 46.4%) (**Fig. 1**).

In our study, decreased visual acuity and field defects were observed in 11 patients (39.3%), all with large tumors and suprasellar extensions.

In our study, conducted on 28 patients diagnosed with GH-secreting pituitary adenomas, 9 microadenomas (32.1%) and 19 macroadenomas (67.9%) of which 3 were GH and PRL mixed secreting pituitary adenomas (10.7%) were identified by using Magnetic Resonance Imaging. Four patients with macroadenomas (14.3%) had various pituitary hormone deficiencies (3 with gonadotropin deficiency and 2 with thyrotrophic deficiency).

From all the macroadenomas reported in the present study, 8 showed cavernous sinus invasion. Tumors invading the cavernous sinus were assessed by using T2 and T1-contrast MRI and classified according to the Knosp grading score, as it follows: 1 patient with Knosp grade 1, 2 patients with Knosp grade 2, 3 patients with Knosp grade 3 and 2 patients with Knosp grade 4.

Regarding the microsurgical approach, the total tumor resection was performed in 18 patients (9 microadenomas (100%) and 9 macroadenomas without extension into the cavernous sinus (47.4%)). The subtotal tumor resection was performed in 10 macroadenomas (52.6%), of which 8 have extended in the cavernous sinus. For the latest ones, a conservative surgical approach was adopted, leaving the intracavernous tumor in situ. In one patient, a hard, solid tumor consistency was discovered intraoperatively, therefore leading to a subtotal resection. Another case showed a midline intracavernous carotid arteries disposition, which led to unsafe conditions of total tumor resection, thus resorting to a subtotal resection.

Gamma Knife postoperative radiosurgery was recommended among patients with postoperative tumor residue, visible on MRI contrast slices. Of the 10 patients who achieved a subtotal resection, only 7 patients underwent Gamma Knife radiosurgery at 3 months postoperatively, whereas 3 patients refused this procedure. Also, medical therapy with Pegvisomant (STH receptor antagonist) was recommended for 7 patients. In 3 patients, who were diagnosed with mixed GH and PRL secreting adenomas, Pegvisomant and Bromocriptine treatment was established.

**Postoperative results**

The resulting rate of endocrinological remission in the group of 28 patients experiencing microsurgical transsphenoidal surgery was 64.3%. Hormone remission rates of microadenomas were 77.8% (7 out of 9 patients) and 57.9% for macroadenomas (11 out of 19 patients).

Visual remission rate in 11 patients with preoperative visual field deficits and decreased visual acuity was 63.6% (7 out of 11 patients). Thus, the visual outcome was positive in 85.7% (24 of 28 patients). No decrease in the visual acuity or visual field was noted postoperatively.

There were no statistically significant age differences between the two types of adenomas (micro- or macroadenomas), that could interfere with the hormonal remission rate (r = 0,235).

Furthermore a correlation between the preoperative serum GH levels and the endocrinological outcome was noticed (r = -0.517), preoperative GH serum levels being significantly lower among patients with complete hormonal remission rate (64.3%). Although there was no correlation between preoperative serum levels of IGF-1 age and sex-matched, and the hormonal remission rate (r = 0,153), the treatment response based on IGF-1 serum levels was continued, because a significant corellation between decreased postoperative serum levels of IGF-1 and longterm hormonal remission rate (r = 0.439) was observed.

**Hormone remission rates based on size and degree of tumor extension**

In the present study, a meaningful correlation was found between tumor invasion in the cavernous sinus and the Knosp grading score (r = 0,498), and also, between intracavernous tumor invasion and tumor size (r = 0.684). Therefore, in our study, large tumors have been associated with intracavernous tumor extension. Likewise, a strong correlation between the cavernous sinus invasion and preoperative GH serum levels was noticed (r = 0.502) in patients with elevated serum levels of GH associating intracavernous tumor extensions. In the present study, no significant statistical correlation was observed between IGF-1 serum levels, for age and sex-matched, and invasion of the cavernous sinus (r = 0,052).

A considerable statistical correlation between the tumor size and hormonal remission rate (r = 0.625) was noticed. Thus, in patients with large tumors, the serum levels of GH and IGF-1 did not suffer a severe drop, requiring drug therapy as an adjuvant treatment. In our study, the tumor sizes were between 0.2 and 5.4 cm (**Table 2**).

**Table 2:** Statistical data concerning tumor size

	*Min*	*Max*	*Mean*	*Median*	*Stddev*
*Diameter (cm3)*	0,2	54,3	26,54286	28,6	15,43453

The best endocrinological outcome was recorded among patients with microadenomas (7 of 9 patients (77.8%)). Additionally, a significant correlation between the degree of tumor resection and the endocrinological outcome was observed (r = 0.598), serum levels of GH and IGF-1 continuing to grow in the presence of a tumor residue. The total tumor resection was achieved in 18 patients, correlated with a hormonal remission rate of 64.3%. Parasellar extension was classified by using the Knosp grading score from 0 to 4, therefore obtaining an average value of 2.75 in our study. Patients with a Knosp grading score between 0 and 2 correlated with a high hormonal remission rate (r = -0.469). **Table 3** provides a list of predictive parameters correlated with a poor endocrinological outcome.

**Table 3:** Variables statistically correlated with a low hormonal remission rate

*Variable*	*Value P*
*Preoperative GH level*	*r = -0.517*
*Tumor size*	*r = 0.625*
*Degree of subtotal resection*	*r = 0.598*
*Knosp 3 and 4 Degree*	*r = 0.469*

In terms of visual impairment, a strong correlation was found between tumor size and preoperative decreased visual acuity (r = 0.714), but also between tumor size and preoperative visual field narrowing (r = 0.698). Similarly, it was noticed that the tumor extension correlated with the degree of preoperative visual impairment (r = 0,547). Regarding the visual outcome, it also correlated with the tumor size (r = 0.658).

**Postoperative complications**

In our study, there were no major complications regarding the 28 patients diagnosed with somatotropinoamas. Postoperative transient diabetes insipidus was observed in one patient, however under treatment with Desmopressin submitted. In another patient, a CSF leak occurred due to a skull base defect, which was submitted 3 days postoperatively by lumbar drainage.

There were no deaths or other postoperative complications. Therefore, morbidity and mortality rates were zero.

## Discussions

The main goal of the surgical approach, in GH-secreting pituitary adenoma, is represented by total tumor resection, within safe conditions, avoiding major postoperative complications and preventing any recurrence [**1**-**3**]. In the multimodal management of acromegaly, first-line therapy is represented by the surgical technique, through microscopic or newly developed endoscopic approach. Gamma Knife radiosurgery and medical therapy are instituted when postoperative hormonal remission is not achieved or when serum hormone levels continue to be elevated due to a tumor residue. Therewith the therapeutic plan aims to control tumor growth, normalize, or reduce hormonal hypersecretion of GH and IGF-1, but also a long-term goal is to reduce the rates of morbidity and mortality, which are much higher in patients with acromegaly compared to the general population [**4**,**5**].

In the last two decades there have been numerous debates regarding the definition of the hormonal remission rate in patients with acromegaly. Starting from 1980, the postoperative serum levels of GH lower than 5µg/ ml represented the major criteria for obtaining a complete hormonal remission, with a surgical success rate of over 75% [**6**]. However, morbidity and mortality rates have remained elevated in the absence of normalized GH and IGF-1 levels [**7**]. Thereby, much stricter criteria were established to define hormonal remission. It was noted that serum GH levels lower than 2 µg/ ml after glucose tolerance test and normalization of serum levels of IGF-1 led to a hormonal remission in 55-70% of the cases of patients [**8**].

In 2000, the Acromegaly Treatment Workshop proposed a precise international criterion, suggesting a complete hormonal remission rate when OGTT suppressed the GH serum levels lower than 1 µg/ L and a normalization of serum IGF-1 levels occurred [**9**-**11**]. In 2005, the Acromegaly Treatment Workshop proposed that suppressed GH serum levels after OGTT lower than 0.4 µg/ L should be considered [**12**]. In 2010, complete hormonal remission criteria were defined: basal serum GH lower than 1 µg/ L, GH levels suppressed after OGTT lower than 0.4 µg/ L and the normalization of IGF-1 serum levels with age and gender-matched.

In our study, the defining criteria chosen for a complete hormonal remission were: normalized serum levels of IGF-1 correlated with age and gender, a basal serum value of GH lower than 2.5 µg/ L, and a level of GH after a glucose tolerance test (OGTT) lower than 1 µg/ L.

Regarding the chosen surgical approach, the long-term hormonal remission rates in GH-secreting pituitary adenomas were different between patients operated via a microsurgical approach and the ones operated via an endoscopic approach. Recent literature studies, performed on GH-secreting pituitary adenomas resected through a microsurgical approach, reported hormone remission rates between 55 to 72.9%, using the criteria from 2000 [**10**,**13**-**15**]. Other studies, using recent criteria from 2010, notified a hormonal remission rate between 42-67% [**7**,**16**-**21**]. Albarel and contributors reported in a study conducted after recent criteria, a hormonal remission rate of 49.5% [**22**]. Cho and Liau [**23**] discovered a hormonal control rate of 73% and a hormonal remission rate of 64% in their series of GH-secreting pituitary adenomas operated microsurgically. Ross and Wilson [**3**] reported, in their study conducted on 1360 GH-secreting pituitary adenomas, a hormonal remission rate of 60.4%. These results corelate with those obtained in our study, namely a hormone remission rate of 64.3% overall, with a rate of 77.8% in microadenomas remission and a remission rate of 57.9% in macroadenoamas. 

Recently, the introducing of endoscopic techniques has represented a major advance in the surgery of pituitary adenomas, providing a safe and effective alternative to the classic microscopic approach. While the endoscopical approach offers more theoretical benefits compared to the standard microsurgical approach, recent literature studies showed no major differences regarding the tumor resection or the postoperative complications rate [**24**]. However, more complex studies should be performed with a longer postoperative follow-up period, to establish some appropriate conclusions. In studies conducted on endoscopic exclusively operated GH-secreting pituitary adenomas, hormonal remission rates between 57-100% were reported [**25**-****36****].

The value varies in terms of the number of patients with suprasellar or parasellar extensions (extension in the cavernous sinus) published in literature. In our study on 28 patients, 9 microadenomas (32.1%) and 19 macroadenomas (67.9%) were reported, of which 8 presented an extension in the cavernous sinus (28.6%).

According to the latest published studies the invasion of the cavernous sinus was between 6-10% [**37**-**39**]. Couldwell [**40**] mentioned that the microscopic transsphenoidal exposure is limited because of the narrow corridor towards the turkish sella, while the endoscopic techniques offer a wider view over the medial and inferior walls of the cavernous sinus, leading to a complete tumor resection. 

Recent studies described a significant correlation between the relatively low hormonal remission and the extension in the cavernous sinus [**41**]. The same hypothesis gathered from the present study, thus obtaining a correlation of r = -0.469. Additionally a significant correlation was observed between the degree of tumor resection and the endocrinological outcome (r = 0.598), therefore serum levels of GH and IGF-1 serum continued to grow in the presence of a tumor residue. The total tumor resection was achieved in 18 patients correlated with a remission rate of 64.3% hormone. 

Likewise, a correlation between the Knosp grading score and the cavernous sinus invasion (r = 0.498) was obtained. In conclusion, a Knosp grade ranging between 0 and 2 correlates with an increased hormonal remission rate (r = 0,498). Moreover, on the opposite side, a Knosp grade of 3 or 4 is correlated with a lower hormonal rate of remission (r = -0,528). Thus, patients with a Knosp grading score of 3 or 4 are generally proposed for medical treatment or radiosurgery to accomplish an effective hormonal control.

Some authors were in favor of implementing the medical therapy as a first line therapy, in case of parasellar tumor extension which led to modifications in the tumors consistency, therefore becoming tough and solid intercepting complete surgical resection [**42**,**43**]. Other authors recommended as a first line therapy the resection of the tumor as much as possible (over 75% tumor volume), afterwards entering drug therapy in order to obtain a more effective control of hormonal remission rate [**44**-**47**].

## Conclusions

After conducting a study on 28 patients diagnosed with GH-secreting pituitary adenomas, a complete hormonal remission was achieved in 64.3% of the patients. Patients with large tumors and parasellar extension in the cavernous sinus, with Knosp grade 3 and 4, with increased serum levels of GH preoperatively, led to subtotal tumor resections, and also to an inefficient hormonal control.

For a complete hormonal remission the defining criteria chosen were: normalized serum levels of IGF-1 correlated with age and gender appropriate, a basal serum value of GH lower than 2.5 µg/ L, and a level of GH after a glucose tolerance test (OGTT) lower than 1 µg/ L. 

In conclusion, a number of predictive factors, which may interfere with the hormonal remission rate from a statistical, clinical and paraclinical point of view were found: tumor size (r = 0.625), preoperative GH serum levels (r = -0.517), cavernous sinus extension quantified according to the Knosp grading score (r = 0.469), and the degree of tumor resection (r = 0.598).

**Acknowlegement**

“This paper was co-financed from the European Social Fund through the Sectoral Operational Programme - Human Resources Development 2007-2013”, project number POSDRU/1871.5/S/155605, entitled “Scientific excellence, knowledge and innovation through doctoral programs in priority areas”, Beneficiary – University of Petrosani.
